# The Application of Reference Dose Prediction Model to Human Health Water Quality Criteria and Risk Assessment

**DOI:** 10.3390/toxics11040318

**Published:** 2023-03-28

**Authors:** Shu-Hui Men, Xin Xie, Xin Zhao, Quan Zhou, Jing-Yi Chen, Cong-Ying Jiao, Zhen-Guang Yan

**Affiliations:** 1 State Key Laboratory of Environmental Criteria and Risk Assessment, Chinese Research Academy of Environmental Sciences, Beijing 100012, China; 2 College of Water Sciences, Beijing Normal University, Beijing 100875, China; 3 China National Environmental Monitoring Center, Beijing 100012, China; 4 Vehicle Emission Control Center, Chinese Research Academy of Environmental Sciences, Beijing 100012, China

**Keywords:** reference dose, molecular descriptor, multiple liner stepwise regression, ambient water quality criteria, health risk assessment

## Abstract

Oral reference dose (RfD) is a key parameter for deriving the human health ambient water quality criteria (AWQC) for non-carcinogenic substances. In this study, a non-experimental approach was used to calculate the RfD values, which explore the potential correlation between toxicity and physicochemical characteristics and the chemical structure of pesticides. The molecular descriptors of contaminants were calculated using T.E.S.T software from EPA, and a prediction model was developed using a stepwise multiple linear regression (MLR) approaches. Approximately 95% and 85% of the data points differ by less than 10-fold and 5-fold between predicted values and true values, respectively, which improves the efficiency of RfD calculation. The model prediction values have certain reference values in the absence of experimental data, which is beneficial to the advancement of contaminant health risk assessment. In addition, using the prediction model constructed in this manuscript, the RfD values of two pesticide substances in the list of priority pollutants are calculated to derive human health water quality criteria. Furthermore, an initial assessment of the health risk was performed by the quotient value method based on the human health water quality criteria calculated by the prediction model.

## 1. Introduction

Water environmental quality criteria are the maximum dose or level of pollutants or harmful factors in the water environment that do not have harmful effects on human health and water ecosystems [1]. The oral reference dose (RfD) is an evaluation metric presented by the US Environmental Protection Agency (EPA) to evaluate the risk of non-carcinogens [2]. It is the estimate of the mean daily dose of exogenous compounds, which is commonly defined as the amount of a chemical to which a person can be exposed on a daily basis over an extended period of time (usually a lifetime) without suffering a deleterious effect. It is an important component of the risk characterization of chemical substances and is also one of the important parameters for the development of water quality criteria for the non-carcinogenic effects of pollutants. RfD was first proposed in a report published by the US Environmental Protection Agency (EPA) in 1988, before which the acceptable daily intake (ADI) was more widely used in the field of toxicology and risk management [3]. Since ADI has some limitations in the field of risk assessment and control, the concept of RfD was introduced to promote consistency in the risk assessment of non-carcinogenic chemicals. The threshold of toxicological concern (TTC) is another important parameter in the field of chemical substance risk assessment. However, the TTC method is not suitable for assessing the safety of chemicals for which toxicological data are required [4].

RfD is an estimate of the average daily exposure dose of exogenous chemicals in environmental media. The two main traditional methods for calculating RfD are the NOAEL/LOAEL method and the benchmark dose method (BMDL) [5,6], and the RfD value is derived by the uncertainty factor UF and the correction factor MF by these traditional methods [7]. These derivation methods require a large investment of time for exposure experiments on mammals [8,9,10]. Since the U.S. EPA issued risk assessment guidelines in the 1980s, RfD values have been obtained for only a few hundred chemical substances [2], so the traditional methods for obtaining RfD are inefficient and constrain the health risk assessment studies of chemical substances. Additionally, the National Science Board proposed in its 2007 report that the study of hazards and risks of contaminants in the environment should make greater use of modern scientific tools and systematic data integration, replacing traditional toxicological methods based on animal experiments [11].

In recent years, a number of studies have used modeling approaches to predict the toxic effects of contaminants [12,13], watershed-scale ecological sensitivity [14], and to achieve toxicity extrapolation among congeners to assess the risk of environmental contaminants [15]. Among these model-building methods, the quantitative structure–activity relationship method is a modeling approach based on the correlation between biological activity and molecular structure, which is widely recognized in the field of toxicology and pharmaceutical research [16,17,18].

In previous studies, NOAEL prediction using chemical SMILES structures, considering only a single kind of descriptors, may ignore the role of certain dominant descriptors [19,20,21]. Toropova built prediction models for NOAEL by SMILES [20]. The R^2^ of the six models ranged between 0.52~0.78. This indicates that these models have poor predictive performance. Moreover, the prediction models developed in some studies only describe the toxic effects of chemicals on some organs, which has some limitations in prediction effects [22]. In addition, when calculating RfD values indirectly by the predicted values of NOAEL and LOAEL, it is difficult to fix the values of uncertainty factors generated by exposure time and experimental animals [23], and the critical endpoints are difficult to define. Although the benchmark dose method makes specific improvements to the NOAEL-based method, it does not address the problems related to non-carcinogenic risk evaluation [24]. Therefore, in the present study, a non-experimental method was considered for the derivation of reference dose values for pesticide-class substances. The toxicity of organic pesticides is closely related to the type and number of functional groups carried by their molecules, in this case, quantitative structure–activity relationship methods may be more effective in the prediction of the physicochemical properties of such substances.

Pesticide poisoning poses a serious threat to aquatic ecosystems. Many of these organisms are highly toxic even at very low concentrations [25]. In China, the rapid development of agriculture and the massive production of pesticides has resulted in the release of large quantities of pesticides into the environment, which are very dangerous due to their extreme toxicity, persistence, and bioaccumulation, posing a major challenge to the safety of ecosystems [26]. Even at concentrations below established lethal thresholds, some pesticides can cause fish kills [27]. For some species, such as carp and salmon, exposure to sublethal concentrations of pesticides can lead to abnormal behavior [27]. In addition, aquatic plants can be endangered or even die under the action of high concentrations of herbicides [28]. Various types of pesticides are currently detected in various environmental media such as water, soil, air, and in animals and humans, and their effects on human health cannot be overlooked [29]. Therefore, pesticide risk assessment and control in China now appear warranted, and the development of local water quality standards for pesticides is urgent.

In order to address the limitations of traditional methods and avoid the interference of uncertainty factors and critical values, this study uses a non-experimental method for predicting the RfD of pesticides directly. The data were collected from a public database called Integrated Risk Information System (IRIS, https://cfpub.epa.gov/ncea/iris/search/ (accessed on 27 July 2022)) and molecular descriptors were calculated based on molecular similarity [30,31]. It fills the data gap of RfD values of chemicals and explores the potential association between toxicity and physicochemical characteristics and chemical structure of pesticides. In addition, the predictive model constructed in this study is used to calculate the RfD values of priority pesticides. The exposure parameters, bioaccumulation coefficients, and other relevant indigenous parameters used to derive the indigenous human health water quality criteria values were determined through the survey data.

## 2. Materials and Methods

### 2.1. Dataset

Quantitative structure–activity relationship models could establish a quantitative relationship between chemical structures and their properties [32]. These computational models are used to predict physicochemical properties of similar compounds that currently lack of experimental data. In this study, the negative log of the reference dose was chosen as the model response value. The source data sets used in this study originate from IRIS, which contains risk information on the cancer and noncancer effects of chemicals, including oral reference dose which depends on the exposure pathway. There are 109 species of pesticide class chemicals that have been included in IRIS which have defined RfD.

The key to obtaining an ideal prediction model is reasonable molecular descriptors. The molecular descriptors of these pesticides were calculated with T.E.S.T. software mentioned by EPA’s official website. This has resulted in 797 descriptors corresponding to 12 descriptor classes. In addition, the screen of the molecular descriptors above was performed by following principles: (1) deleting the descriptors that a variance of 0; (2) deleting the descriptors that have a number of non-zero values less than 10%; (3) deleting one of two descriptors that the correlation coefficients greater than 0.90. After the above pre-processing, 372 descriptors remained for the prediction analysis.

### 2.2. Model Building

The preprocessed set of molecular descriptors was used as the independent variable X, and the negative log of the reference dose RfD value (−logRfD) was used as the dependent variable Y. The multivariate stepwise linear regression method in SPSS software (version 26.0, IBM Inc. Chicago, IL, USA) was applied to establish the regression model between molecular descriptors and −logRfD. Moreover, the variance inflation factor (VIP) was used to verify whether there was multicollinearity among the descriptors in the model, and Durbin–Watson values (D-W) were used to test the model autocorrelation. In the total data set, 70~80% were randomly selected for the training set and 20~30% for the test set. Internal validation and external validation were used to verify the predictive ability and robustness of the model. The model was also used to predict the RfD of EPA-released priority pesticides lacking RfD values.

### 2.3. Derivation of Human Health Water Quality Criteria

This study focuses on the non-carcinogenic effects of p-p’DDE and α-HCH, and the human health water quality criteria are derived according to the *Technical Guideline for Deriving Water Quality Criteria for the Protection of Human Health* [33]. The human health ambient water quality criteria (AWQC) for non-carcinogenic effect is calculated according to the following equation:

(1) $$\mathrm{AWQC}=\mathrm{RfD}\cdot \mathrm{RSC}\cdot \left(\frac{\mathrm{BW}}{\mathrm{DI}+\sum_{i=2}^{4}\left({\mathrm{FI}}_{i}\cdot {\mathrm{BAF}}_{i}\right)}\right)$$

where RfD is the reference dose (mg · kg^−1^ · d^−1^) for non-carcinogenic effects; RSC is the relative source contribution to account for non-source exposures; BW is body weight (kg); DI is drinking water intake (L · d^−1^); FI_i_ is intake of aquatic products (kg · d^−1^) for each trophic levels (i = 2, 3, 4); BAF_i_ is the bioaccumulation factor (L · kg^−1^) for each trophic level (i = 2, 3, 4).

The RfD values were adopted from the predicted values of the predictive model constructed in this study, and the rest of the relevant parameters required for the derivation of water quality criteria for human health were referred to the relevant data in the *Exposure Factors Handbook of Chinese Population (Adult Volume)* [34] and the *Nutrition and Dietary Guidelines for Chinese Residents* [35]. In addition, both p-p’DDE and α-HCH are non-ionic organics, and the bioaccumulation factors were determined by using laboratory BCF and food chain multiplication factors with reference to the derivation method of bioaccumulation factors in the technical guideline and the framework of derivation method selection in human health methodology [6]. The baseline BAF level final trophic level BAF is calculated as follows:

(2) $${\mathrm{BL}}_{\mathrm{BAF}}=\mathrm{FCM}\cdot \left(\frac{\mathrm{BCF}}{f_{\mathrm{fd}}}-1\right)\cdot \frac{1}{f_{l}}$$

(3) $$F_{\mathrm{BAF}}=\left(\mathrm{BL}\_\mathrm{BAF}\cdot f_{l}+1\right)\cdot f_{\mathrm{fd}}$$

where BCF is the bioconcentration factor (L · kg^−1^); FCM is the food chain multiplication factor; f_l_ is the fraction of lipids in biological tissues; and f_fd_ is the fraction of free dissolved state of the chemical in the aqueous environment, which is calculated as follows:

(4) $$f_{\mathrm{fd}}=\frac{1}{1+\mathrm{POC}\cdot K_{\mathrm{ow}}+\mathrm{DOC}\cdot 0.08K_{\mathrm{ow}}}$$

where POC is the concentration of particulate organic carbon in water (kg · L^−1^); DOC is the concentration of dissolved organic carbon in water (kg · L^−1^); K_ow_ is the octanol-water partition coefficient of the chemical.

### 2.4. Health Risk Assessment

The quotient method was used in this study to evaluate the health risks of p-p’DDE and α-HCH in the aqueous environment with the following equations:

(5) HQ=EEC/AWQC

where EEC is the environmental exposure concentration in the water environment; AWQC is the human health water quality criteria. According to the size of the HQ value, the potential risk of pollutants can be divided into the following levels: HQ < 0.1000, no risk; 0.1000 ≤ HQ ≤ 1.000, there is a low risk; 1.000 ≤ HQ ≤ 10.00, there is an intermediate risk; HQ > 10.00, there is a high risk.

## 3. Results and Discussion

### 3.1. Prediction Models for Pesticide Class Chemicals

The 109 molecules were randomly divided into a training set and a test set containing 80 and 29 molecules, respectively. The predictor variables were selected among the remaining 372 molecular descriptors after the primary screening, and multiple stepwise regression analysis was performed to build a model for the training set, and the test set was used as an external validation to evaluate the predictive ability of the model. Figure S1 shows the relationship between R_adj_^2^ and the number of molecular descriptors used to determine the number of descriptors in the model to prevent model overfitting.

The optimal MLR model and the descriptor obtained are shown in the following equation:

−LogRfD = 1.468 − 0.483 × Ui + 0.361 × ATS1m − 0.195 × MAXDP + 0.265 × xp9 − 0.312 × SdssC_acnt + 1.516 × ssi − 0.108 × SHHBd − 0.559 × MATS8e − 1.162 × MATS2m + 0.76 × MATS2e + 0.097 × SsssCH_acnt + 0.108 × piPC08

The meaning of each descriptor is shown in Table 1.

**Figure 1:**
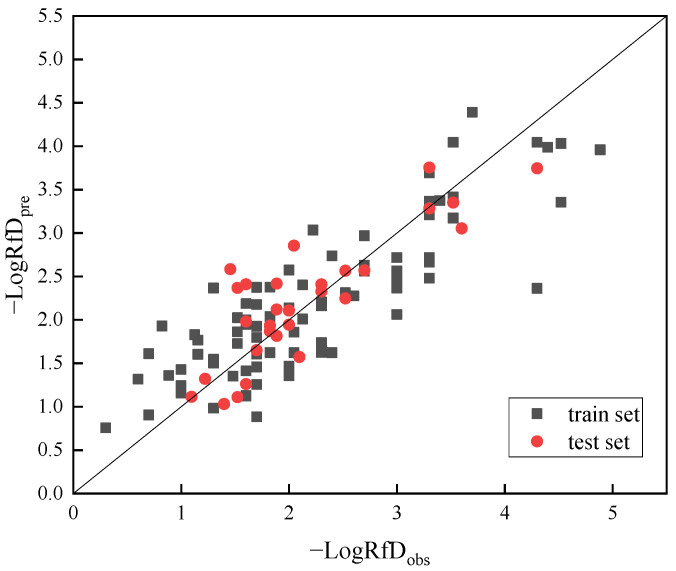
Graphical representation of predicted −logRfD versus observed −logRfD. The squares refer to data in training set and the dots are data in test set. The actual and predicted values of the negative logarithm of RfD are the abscissa and ordinate, respectively.

Based on the results of the *t*-test, it is clear that the descriptor Ui contributes the most to the model and is the most important molecular descriptor associated with pesticide RfD. The VIP values of all independent variables in the model were less than 5, indicating low autocorrelation among the respective variables, and therefore the descriptors were chosen reasonably. As can be seen from the information in Figure 1 all data points are relatively evenly distributed around the diagonal line for both the training set samples and the test set samples, with no particularly obvious outliers, indicating that the model has a good fitting estimation ability for the training samples and good prediction ability for the external compounds.

The statistical parameters of the MLR model are shown in Table 2. The results of R_tra_^2^ = 0.762, and *p* < 0.05, indicate that the model built by the selected descriptors has a good fit. Additionally, the Durbin–Watson test (D-W test) is the most commonly used method to test the autocorrelation of the model [36]. The closer the DW value is to 2, the less autocorrelation there is in the model, and the model is acceptable when 1.5 < DW < 2.5. In this study, the DW value (1.952) indicates that the correlation between the descriptors and the model is good. As indicated by the external validation results, R_tes_^2^ = 0.683 and RMSEP = 0.434, which indicates that the model has good stability and good external prediction ability. The cross-validation could be used for describing the fitting effect on the training set, and cross-validation correlation coefficients (q^2^) are expected to be greater than 0.5. The dataset modeled in this paper contains only 109 compounds, so it is not suitable to divide the independent validation set. Consequently, the hold-out cross-validation method was chosen to evaluate the validity of the model performance. To further verify the reliability of the model, the validation method proposed by Roy for external testers is used, and k = 0.983 > 0.88 and k’ = 1.016 < 1.15 are obtained, which satisfy the corresponding validation requirements [37]. This indicates that there is no systematic error in the model itself that would cause the prediction results to deviate in a particular direction. The combination of the above results indicates that the predictive ability of the model is acceptable.

**Figure 2:**
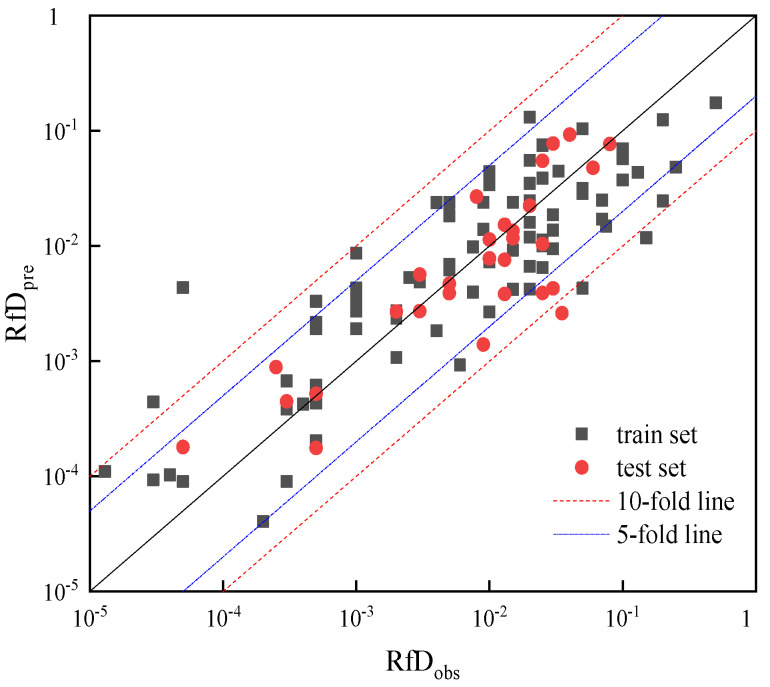
Comparison of observed and MLR-predicted RfD. The squares refer to data in training set and the dots are data in test set. The solid line represents the 1:1 line, while dot-dash lines and short-dashed lines represent a 5-fold and 10-fold difference, respectively, between these values.

The relationship between the predicted and actual values of −log RfD obtained from the MLR model is shown in Figure 1. Comparing the actual RfD values with those predicted by the model (Figure 2), it can be seen that for the vast majority of pollutants (>95%), the difference between the true and predicted values is within a factor of 10, and for most (>85%) pollutants the difference between the actual and predicted values is within a factor of 5. Consequently, the consistency between predicted and actual values also proved the accuracy of the predictive models.

The predicted RfD values of the two pesticides mentioned are 0.01271 mg · kg^−1^ · d^−1^ and 0.0002124 mg · kg^−1^ · d^−1^, respectively, which are obtained by the above equation with the corresponding molecular parameters. From the biological conception, the RfD values of these pesticides in this manuscript are identified as 0.01 mg · kg^−1^ · d^−1^ and 0.0002 mg · kg^−1^ · d^−1^.

### 3.2. Determination of Other Water Quality Criteria Parameters

The lipid fraction values were calculated using the average value of lipids of each species as the default value. The lgK_ow_ value of p-p’DDE is 6.76 and the lgK_ow_ value of α-HCH is 3.69, which obtained from the data disclosed on the official website of EPA. From Equation (4), the free dissolved state of p-p’DDE and α-HCH are 0.0319 and 0.9753, respectively.

According to the derivation steps of non-ionic organic compound bioaccumulation factors in the technical guideline, the baseline bioaccumulation factors and final trophic level bioaccumulation factors were calculated for different trophic levels, and the required parameter values and calculation results are shown in Table 3.

In the water quality criteria derivation formula, the human body weight BW and daily water intake DI refer to the handbook mentioned above published by the Ministry of Environmental Protection in 2013. The average body weight of adults over 18 years old in China is 60.6 kg, and the daily water intake is 1.85 L · d^−1^. Moreover, the intake of water products at each trophic level refers to the recommended values in the Nutrition and Dietary Guidelines for Chinese Residents [35]. The relative source contribution rate was taken with reference to the exposure decision tree method in the technical guidelines, and finally, the 20% default value was used as the RSC value in this study. The RfD value was the predicted value of the model constructed in this manuscript. The localized parameters required to calculate the human health water quality criteria were shown in Table 4. After calculating by Eq 1, the human health water quality criteria of p-p’DDE and α-HCH are 0.03 μg · L^−1^ and 0.02 μg · L^−1^, respectively.

### 3.3. Health Risk Assessment

Organochlorine pesticides are highly fat-soluble and can enter the human body and animals through the food chain and can accumulate in the visceral tissues. Therefore, the health risk caused by organochlorine pesticides is of concern. In this study, the risk assessment of p-p’DDE and α-HCH in a domestic water environment was performed by the Hazard Quotient method (HQ) of Equation (5). The exposure concentrations used are the publicly released survey data in recent years, involving 192 and 254 sampling locations, respectively. In addition, the specific information is shown in Supplementary Table S1. The exposure concentration of p-p’DDE at the sampling sites ranged from 0.002 to 139 ng/L, with 49% of the sites having HQ values less than 0.1000; 18% of the sites had HQ values between 0.1000 and 1.000; and 33% of the sites had HQ values between 1.000 and 10.00. The exposure concentration of α-HCH at each sampling site ranged from 0.0151 to 297 ng/L, 60% of the sites had HQ values less than 0.1000; 36% of the sites had HQ values between 0.1000 and 1.000; and 4% of the sites had HQ values between 1.000 and 10.00. The median values of the monitored concentrations were used to represent the exposure levels of pollutants in the domestic water environment, and the HQ values of the two pollutants were calculated to be 0.13 and 0.08, respectively. The results show that at the current exposure levels, α-HCH basically poses no health risk to the residents around the watershed, and p-p’DDE poses a lower health risk to the residents. Based on the potential human health risks of organochlorine pesticides, it is necessary to control the pollution problems in the corresponding areas to ensure the water safety of the residents in these areas.

## 4. Conclusions

Currently, the development of water quality criteria and risk assessment of pesticide compounds is an issue of concern. Moreover, RfD is a key parameter of water quality criteria derivation. In this paper, through a non-experimental method, the RfD prediction model was constructed using molecular descriptors for the derivation of human health water quality criteria values. In the absence of experimental data, the predicted value of the model has a certain reference value, which is conducive to the advancement of pollutant health risk assessment.

The model obtained in this paper has good model stability in terms of statistics (Rtra2 = 0.762, Rtes2 = 0.683, RMSEP = 0.434). In a previous study, Mazzatorta used MLR to build prediction models for LOAEL [21], which had 15 descriptors (R2 = 0.50, RMSE = 0.727). Consequently, the predictive model for RfD inheres has higher reliability. In addition, some researchers used the QSAR approach to model the extrapolation of toxicity between BETX [15], and 81% of the species had a prediction error of 10 times or less. In this study, for the vast majority of pollutants (>95%), the difference between the true and predicted values is within a factor of 10, and for most (>85%) pollutants the difference between the actual and predicted values is within a factor of 5. In summary, the RfD prediction model in this paper has higher reliability.

The human health water quality criteria of p-p’DDE and α-HCH based on localized parameters are 0.03 μg · L^−1^ and 0.02 μg · L^−1^, respectively. Moreover, the quotient method was used to make a preliminary evaluation of the health risks of p-p’DDE and α-HCH in the water environment. The results demonstrated that, under the current exposure level, p-p’DDE is basically no health risk to the residents around the watershed, and α-HCH produces a lower health risk to the residents. This result may be due to the fact that p-p’DDE has a higher bioaccumulation factor and is therefore potentially more hazardous to human health.

Although there are important discoveries revealed by these studies, there are also limitations. Since there are only about 300 compounds with clearly defined RfD values in the IRIS system, even fewer pollutants meet the requirements of this study. Consequently, the dataset used in this paper contains only 109 compounds, which is a limited sample size. Therefore, if more compounds are added to the IRIS system in the future, this study should increase the sample size to optimize the model.

## Figures and Tables

**Table 1:** The concept of different descriptors included in model.

No	Descriptor	Description
1	Ui	Unsaturation index
2	ATS1m	Broto–Moreau autocorrelation of a topological structure—lag 1/weighted by atomic masses
3	MAXDP	Maximal electrotopological positive variation
4	xp9	Simple 9th order path chi index
5	SdssC_acnt	Count of (=C<)
6	ssi	Standardized Shannon Information or standardized information content
7	SHHBd	Sum of E-State indices for hydrogen bond donors
8	MATS8e	Moran autocorrelation—lag 8/weighted by atomic Sanderson electronegativities
9	MATS2m	Moran autocorrelation—lag 2/weighted by atomic masses
10	MATS2e	Moran autocorrelation—lag 2/weighted by atomic Sanderson electronegativities
11	SsssCH_acnt	Count of (>CH–)
12	piPC08	Molecular multiple path count of order 08

**Table 2:** The description and statistical information of the predictive model.

*N*	R_tra_^2^	R_tes_^2^	RMSEP	*p*	D-W	q^2^	k	k’
12	0.762	0.683	0.434	<0.05	1.952	0.648	0.983	1.016

**Table 3:** Bioaccumulation factor parameters and calculated values.

Trophic Levels	f_l_	Compounds	FCM	BL-BAF	F-BAF
2	0.019	p-p’DDE	1.000	5.33 × 10^7^	3.24 × 10^4^
		α-HCH	1.000	1.97 × 10^4^	365
3	0.026	p-p’DDE	13.30	5.18 × 10^8^	4.30 × 10^5^
		α-HCH	24.70	3.55 × 10^5^	9.00 × 10^3^
4	0.030	p-p’DDE	1.128	3.81 × 10^7^	3.65 × 10^4^
		α-HCH	1.003	1.25 × 10^4^	366

**Table 4:** Statistical table of human health water quality parameters.

Compounds	RfD	BW	DI	FIi/kg · d^−1^			BAF/L · kg^−1^		
	mg · kg^−1^ · d^−1^	kg	L · d^−1^	FI_2_	FI_3_	FI_4_	2	3	4
p-p’DDE	0.01	60.60	1.850	0.0126	0.0100	0.0075	3.24 × 10^4^	4.30 × 10^5^	3.65 × 10^4^
α-HCH	0.0002						365	9.00 × 10^3^	366

## Data Availability

No new data were created or analyzed in this study. Data sharing is not applicable to this article.

## Supplementary Materials

The following supporting information can be downloaded at: https://www.mdpi.com/article/10.3390/toxics11040318/s1, Figure S1: The relationship between R_adj_^2^ and the number of molecular descriptors; Table S1: Parameters of health risk assessment in water environmental. References [38,39,40,41,42,43] are cited in the Supplementary Materials.
