# Supplementary material for: The Application of Reference Dose Prediction Model to Human Health Water Quality Criteria and Risk Assessment
Source: Toxics. 2023 Mar 28;11(4):318. doi: 10.3390/toxics11040318 (PMC10146768; doi:10.3390/toxics11040318)
Supplement: Supplementary file 1 [file toxics-11-00318-s001.zip › toxics-2178581-supplementary.pdf]

Table S1. Parameters of health risk assessment in water environmental

compounds	n	Exposure concentration (ng/L)		HQ value	AWQC (μg/L)	References
		range	median			
p-p'DDE	192	0.0020~138.9	3.950	0.1279	0.03	[38-43]
α-HCH	254	0.0151~297.0	2.200	0.0848	0.02	

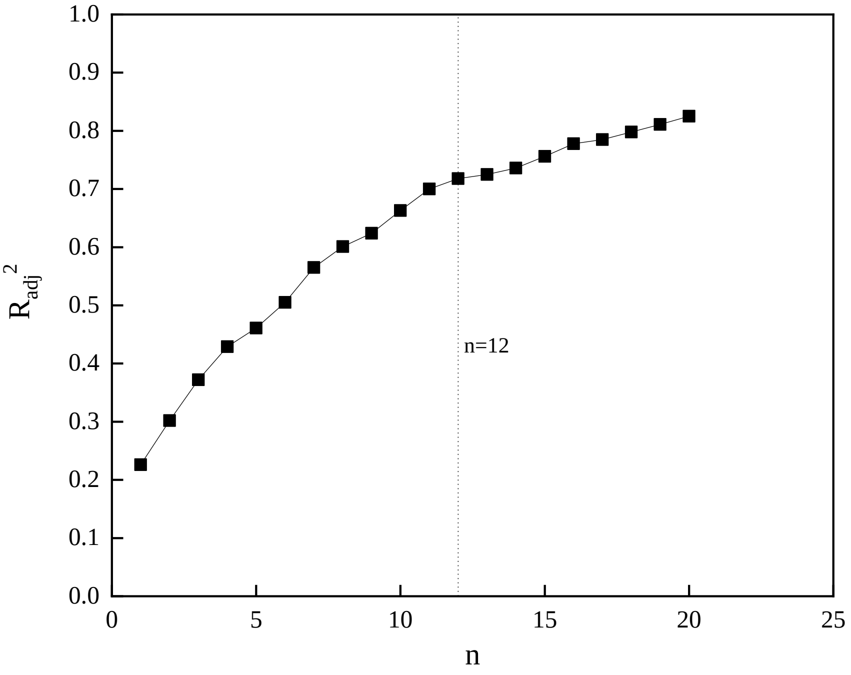

Figure S1. The relationship between R_{adj}^2 and the number of molecular descriptors
